# Dissociation of feeling and belief in the rubber hand illusion

**DOI:** 10.1371/journal.pone.0206367

**Published:** 2018-10-23

**Authors:** Luigi Tamè, Sally A. Linkenauger, Matthew R. Longo

**Affiliations:** 1 Department of Psychological Sciences, Birkbeck, University of London, London, United Kingdom; 2 Department of Psychology, Lancaster University, Lancaster, United Kingdom; University of Exeter, UNITED KINGDOM

## Abstract

The Rubber Hand Illusion (RHI) has been widely used to investigate the perception of the bodily self. Commonly used measures of the illusion are self-report questionnaires and proprioceptive drift of the participants’ hands towards the rubber hand. Recent studies have shown that these measures can be dissociated, suggesting they may arise from distinct mechanisms. In previous studies using questionnaires, participants were asked to base responses on their subjective feelings of body ownership, rather than their beliefs. This makes sense given the obvious fact that whereas participants may *feel* like the rubber hand is part of their body, they do not *believe* that it is. It is not clear, however, whether a similar dissociation between feelings and beliefs also exists for proprioceptive drift. Here, we investigated the presence of a dissociation between feeling and belief in the context of the RHI. When participants reported their feelings there was an increase both in the sense of body ownership over the fake hand as well as in the proprioceptive drift, compared to when they reported their beliefs. Strikingly, unlike the sense of ownership, proprioceptive drift was unaffected by the synchrony of stimulation. This may be an important way in which the two measures of the RHI differ.

## Introduction

The way we represent our body strongly relies on the integration of information from different sensory modalities [1]. A classic example of this type of integration is the Rubber Hand Illusion (RHI) [2]. In this paradigm, participants observe a rubber hand being stroked while their unseen real hand is synchronously touched. After synchronous stimulation, participants tend to perceive the location of their own occluded hand misplaced toward the rubber hand [3]. Moreover, they report feeling the tactile sensation originating from the rubber hand, as if they could experience touch through it [4], which sometime results in a feeling of ownership over the fake hand [5].

While a range of measures have been used to quantify the illusion, the two most widely used are a questionnaire assessing the subjective experience of the illusion, and proprioceptive misplacement of the participants’ own hand towards the rubber hand, which is typically considered as more objective and resistant to demand characteristics. Early work suggested that these two measures reflect a common underlying mechanism [2]. More recently, however, some studies in healthy individuals [6,7] and patients [8] have shown that they can be dissociated, suggesting that they may arise from different underlying processes [9].

Here, we consider an aspect of the RHI which has not been a focus of research, namely the obvious fact that while participants report *feeling* like the rubber hand is part of their body, they do not *believe* that it really is. There is a rubber hand *il*lusion, and not a rubber hand *de*lusion. Accordingly, questionnaires assessing the subjective experience of the illusion have naturally used ‘apparent’ phrasing, asking participants about what it “felt like” during the illusion, rather than what they “really thought” was happening [2,5]. Interestingly, it is not obvious whether there is an analogous dissociation between feeling and belief in the proprioceptive drift induced by the illusion. Participants may be genuinely fooled about the location of their hand, in a way that they are not fooled about the attribution of ownership over the rubber hand. Indeed, studies have been inconsistent in their phrasing to participants about proprioceptive localisation. For example, Tsakiris and Haggard [3], asked participants “Where is your index finger?”, a phrasing which suggests a judgment of where the finger actually is. In contrast, Longo and colleagues [5] asked participants to judge “where it felt like the tip of their index finger was located”. No studies have compared these types of instructions, making it unclear whether there is a dissociation between feeling and belief for proprioceptive drift, as there obviously is for the subjective experience of body ownership. Consequently, the dissociation between the drift and questionnaire measures may tap into different constructs that are differently affected by instructions. This may be an important way in which these measures differ, perhaps accounting for lack of correlation between the feeling of ownership and proprioceptive drift [7].

A few studies have investigated the potential dissociation between feeling and belief in the context of body perception. Ekroll and colleagues [10], using a visuo-proprioceptive illusion (the ‘shrunken finger illusion’), found that participants’ finger length underestimation was about four times larger when asked about their feeling rather than their belief. Hence, Ekroll and colleagues suggested that the illusory experience of a ‘shrunk’ finger maybe based on perceptual rather than cognitive processing. Yet, this dissociation is not always present. Tamè and colleagues [11] found no influence of instruction when studying several types of distorted body representations. However, these distortions were pre-existing and intrinsic to the body rather than needing to be elicited using a manipulation as in the shrunken finger illusion. Thus, it remains unclear whether instructions about *feelings* rather than *beliefs* in the context of a RHI paradigm, can affect proprioceptive localization. This provides a potentially novel way of dissociating the underlying mechanisms producing these aspects of the RHI; participants may be genuinely deluded about the location of the finger, while being merely illuded about the experience of body ownership. Hence, the current study investigated dissociation between *feeling* and *belief* instructions in the RHI. We used a standard RHI paradigm measuring subjective reports and proprioceptive drift, but asked participants to adopt different attitudes towards their judgments.

## Materials and methods

### Participants

Twenty people (*M* ± *SD* = 28.5 ± 6.9 years; 13 females) participated. Participants gave informed consent and reported normal or corrected-to-normal vision and touch. The study was performed in accordance to the Declaration of Helsinki and approved by the Departmental Ethics Committee of the Department of Psychological Sciences, Birkbeck College, University of London. Written informed consent was obtained from all participants. Seventeen participants were right-hand, as assessed by the Edinburgh Handedness Inventory (*M* = 65, range -100-100, [12]).

### Design

The experiment had a repeated-measures design with two factors: instruction (Feeling, Belief) and stimulation (Synchronous, Asynchronous). The four blocks were randomised and repeated three times with a different order, resulting in twelve blocks overall.

### Procedure

**Fig 1:**
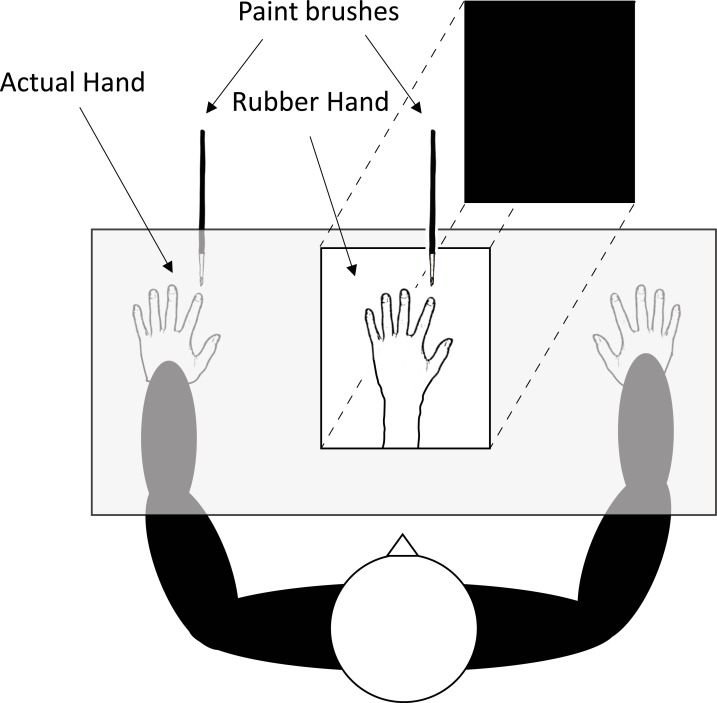
Experimental set-up for the rubber hand illusion.

The participant was seated at a table opposite the experimenter, with their left arm placed through an entrance hole and resting in a specially constructed box (75 cm x 59 cm x 17 cm; see Fig 1). A life-size model of a left hand and forearm was placed in the box, aligned with the participant’s body midline. The participant could see this fake hand through a window in the box. The box had a hinged cover which when lifted exposed the fake hand and hid the experimenter from view. Participants wore a cloth smock that was attached to the front of the box and hid their real arm from view. The distance between the participant’s index finger and the index finger of the fake hand was 22 cm. The back of the box was removed to allow the experimenter to access the participant’s hand and the fake hand.

Two visuotactile induction conditions, synchronous and asynchronous, were performed. At the beginning of each trial, the cover of the box was raised and the participant was instructed to focus on the rubber hand while two paintbrushes stroked the index fingers of the fake hand and the participant’s real hand for 60 s at approximately 1 stroke/s. In the synchronous condition, the timing of the brush strokes was synchronized, whereas in the asynchronous condition the timing of the brush strokes was 180° out of phase. Before each trial, two finger location judgements were obtained by placing a ruler across the top of the box and asking participants to indicate where they felt/believed, depending on the condition, the tip of their left index finger was located. The placement of this ruler varied from trial to trial to prevent participants repeating responses in subsequent trials. After stimulation, the box cover was lowered and two post-induction finger location judgements were obtained in the same manner as before the induction.

In different blocks, the experimenter asked participants to adopt different approaches to the task. At the beginning of the study, the following description of the two approaches was given:

*“In different blocks of trials, I’ll ask you to adopt different approaches to the task both for the localization of your finger and the questionnaire. On some blocks, I’ll ask you to judge the location where it FEELS like your left index finger is located and to respond to the questionnaire according to your subjective experience. In this case, we’re interested in your subjective experience of the location of your finger and subjective percept. On other blocks, I’ll ask you to judge the location where you think your left index finger REALLY IS and to respond to the questionnaire according to your objective beliefs. In this case, we’re interested in your objective beliefs about the location of your finger and objective percept*.*”*

**Fig 2:**
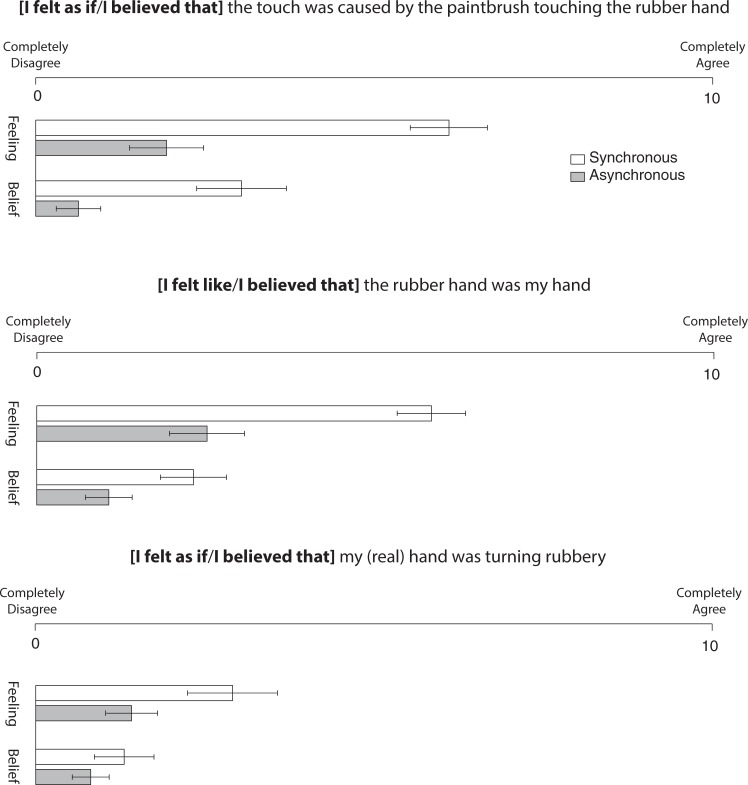
Questionnaire administered to participants after each trial. On each question, participants were allowed to give any number between minimum level and maximum level [5]. Error bars represent the Standard Error of the Mean (±SEM).

Fig 2 shows a short questionnaire that was administered verbally after each trial to assess the participant’s feeling or belief experience. Items in the questionnaire were slightly different depending of the instruction condition. The questionnaire consists of 3 items, two of interest and a third as a control for demand characteristics. Participants rated their agreement on a scale ranging from 0 (completely disagree) to 10 (completely agree).

### Data analysis

Finger location judgement was calculated as the difference between the position reported by the participant and the actual position of the participant’s finger. A positive value indicates a judgement to the right of a participant’s actual finger location (i.e. towards the fake hand) and a negative value indicates a judgement to the left of the actual finger location (i.e. away from the fake hand). Proprioceptive drift was calculated by subtracting the pre-induction finger location judgement from the post-induction finger location judgement. The proprioceptive drift values were entered into a repeated measures Analysis of Variance (ANOVA) with instruction (Apparent, Objective) and stimulation (Synchronous, Asynchronous) as within-participant factors. Moreover, values from the questionnaire for each question were entered into three separate ANOVAs with the same factors, specifically instruction and stimulation. Two-tailed paired t-tests were used for all planned comparisons. The raw data are publicly available at https://osf.io/9kw2c/.

## Results

### Questionnaire

For question 1 (touch), an ANOVA revealed significant main effects of instruction, *F*(1,19) = 47.34, *p* = .0001, *MSE* = 2.01, *η*_*p*_^*2*^ = .71, and stimulation, *F*(1,19) = 31.96, *p* = .0001, *MSE* = 6.77, *η*_*p*_^*2*^ = .63, which were modified by an interaction, *F*(1,19) = 6.12, *p* = .023, *MSE* = 2.54, *η*_*p*_^*2*^ = .24. Participants more strongly agreed with the statement with the *feeling* instruction when they were stimulated synchronously (*M*±*SE* = 6.1±0.6) compared to asynchronously (*M*±*SE* = 1.9±0.5; *t*(19) = 5.40, *p* = 0.0001, *d*_*z*_ = 1.21). This dissociation was present also when they were performing the task under the *belief* instruction, however, it was less prominent (*t*(19) = 4.20, *p* = 0.001, *d*_*z*_ = 0.93). As expected this result shows a dissociation between the synchronous and asynchronous conditions, more interestingly, it shows a dissociation between the types of instruction adopted.

An ANOVA on the data for the question 2 revealed significant main effects of instruction, *F*(1,19) = 32.67, *p* = .0001, *MSE* = 3.77, *η*_*p*_^*2*^ = .63, and stimulation, *F*(1,19) = 55.77, *p* = .0001, *MSE* = 1.87, *η*_*p*_^*2*^ = .75, which were subsidiary to an interaction, *F*(1,19) = 15.61, *p* = .001, *MSE* = 1.36, *η*_*p*_^*2*^ = .45. As for the previous item, participants more strongly agreed with the statement with instruction about their *feeling* when they were stimulated synchronously (*M*±*SE* = 5.8±0.5) compared to asynchronously (*M*±*SE* = 2.5±0.6; *t*(19) = 6.55, *p* = .0001, *d*_***z***_ = 1.46). This dissociation was present also when they were performing the task with instruction about their *belief*, however, it was less prominent (*t*(19) = 4.83, *p* = .0001, *d*_***z***_ = 1.10).

Finally, as shown in bottom of Fig 2 an ANOVA on the participants’ rating for question 3 revealed a significant main effect of instruction, *F*(1,19) = 7.44, *p* = .013, *MSE* = 3.26, *η*_*p*_^*2*^ = .28, caused by the fact that participants more strongly agreed with the items in the *feeling* (*M*±*SE* = 2.2±0.5) compared to the *belief* (*M*±*SE* = 1.1±0.3) instruction. Moreover, there was also a significant main effect of stimulation, *F*(1,19) = 9.98, *p* = .005, *MSE* = 1.98, *η*_*p*_^*2*^ = .34, caused by the fact that participants more strongly agreed with the item in the synchronous (*M*±*SE* = 2.1±0.5) compared to the asynchronous (*M*±*SE* = 1.1±0.3) stimulation condition. However, the interaction between instruction and stimulation was only marginally significant, *F*(1,19) = 3.99, *p* = .06, *MSE* = 1.24, *η*_*p*_^*2*^ = .17.

Overall, these results indicate that there were significant differences in the perceived illusion strength between the synchronous and asynchronous conditions. Critically, this difference was more prominent when participants were instructed to respond about their feeling than their belief.

### Proprioceptive drift

**Fig 3:**
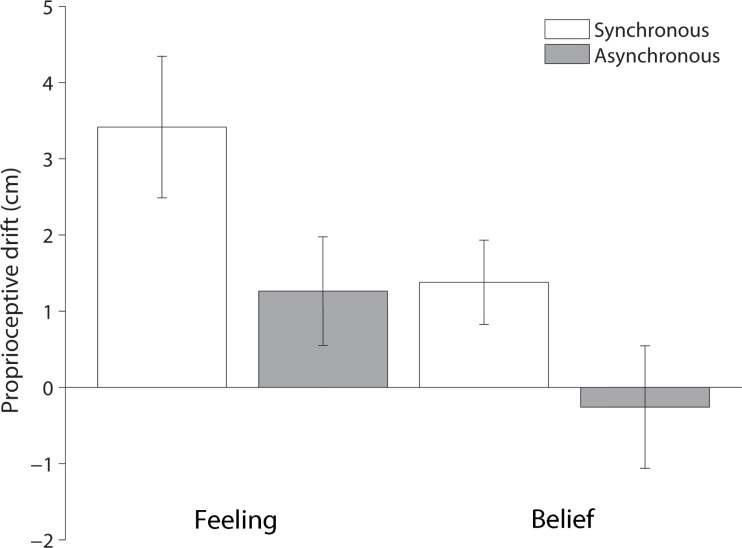
Mean proprioceptive drift towards the rubber hand for the synchronous and asynchronous stimulation in the *Feeling* and *Belief* instruction conditions. Error bars represent 95% within-subjects Confidence Intervals.

An ANOVA on the drift measurement revealed a significant main effect of instruction, *F*(1,19) = 14.77, *p* = .001, *MSE* = 4.28, η_p_^2^ = .44. As shown in Fig 3, participants had a greater proprioceptive drift towards the rubber hand when instructed to respond about their *feeling* (*M*±*SE* = 2.3±0.6cm) compared to their *belief* (*M*±*SE* = 0.6±0.3cm), see Fig 3. Moreover, there was a main effect of stimulation, *F*(1,19) = 10.58, p = .004, *MSE* = 6.79, *η*_*p*_^*2*^ = .36, caused by a greater proprioceptive drift for the synchronous (*M*±*SE* = 2.4±0.6cm) compared to the asynchronous (*M*±*SE* = 0.5±0.3cm) stimulation condition. However, in contrast to the questionnaire, there was no significant interaction between these two factors, *F*(1,19) = 1.32, *p* = .27, *MSE* = 1.01, *η*_*p*_^*2*^ = .07.

## Discussion

Here, we aimed to investigate the potential dissociation between participants’ *feeling* and *belief* in the context of the RHI. We were particularly interested if such a dissociation was present for the proprioceptive drift measure. We replicated the classic results in the RHI literature in which participants experience a stronger illusion when they are stimulated synchronously than asynchronously in both measures, supporting the suitability of our approach. Our main finding is that when participants are asked to report their *feelings* they report having a stronger illusion compared to when they are asked to report their *beliefs*, as shown by a greater sense of body ownership and–more critically–by a larger proprioceptive drift.

For the proprioceptive drift measure, we did not find an interaction between instruction and stimulation conditions. Hence, when participants were looking at the rubber hand they may have been more genuinely fooled about the location of their real hand when asked to respond about their feeling than their belief regardless of the fact that stimulation was synchronous or asynchronous. This is an interesting effect considering that typically the asynchronous stimulation is not meant to induce the illusion of experiencing the rubber hand as your own and is apparent also in a reduced proprioceptive drift and questionnaire compared to the synchronous stimulation. A possible reason for this pattern of results is that participants were experiencing the illusion merely by seeing the rubber hand while their own hand was hidden. It has been previously shown that vision itself can induce a proprioceptive drifts comparable to the synchronous stimulation condition [7,13]. Our effects for proprioceptive drift could result from the possibility that when participants responded about their feeling, they may have reduced their “rational” resistance to the illusion. This process probably occurs before participants are stimulated, as a mental state that is carried on during the synchronous and asynchronous stroking. Therefore, the participants’ attitude to respond, about their feeling or belief, when somehow set a priori at the beginning of the block is then maintained across trials unaffected by the type of stimulation. In this respect, Rohde and colleagues [7], have shown that with frequent measurements the proprioceptive drift occurs not only in the synchronous condition, but also with asynchronous stroking and when just vision of the hand is present without any stroking. On the basis of their data, authors suggested that prolonged synchronous stroking causes a proprioceptive drift in the RHI, however, this drift is already present before the stroking is applied. By contrast, the prolonged asynchronous stroking is interfering with the proprioceptive drift by interfering with the visuo-proprioceptive integration of the visual location of the rubber hand and the proprioceptive signal coming from the real hand.

Interestingly, the proprioceptive drift was influenced by instruction type. Drift reduced when participants were instructed to indicate where they believed their hand to be located as opposing to where they felt their hand be located. Indeed, the magnitude of the drift in the feel asynchronous condition was the same as the magnitude of the drift in the synchronous belief condition. If proprioceptive drift can be influenced by the attitudes of the participant, then the experimental hypothesis may not be as opaque to participants as commonly thought [7,14]. If drift is not an objective measure, then it is unclear what proprioceptive drift is actually measuring and how it is different from subjective reports. It could very well be that the drift measure is not actually a measure of the illusion, but rather a result of demand. It would be interesting to see if the physiological measures associated with the illusion, which are also considered objective, such as skin temperature and galvanic skin response could be modulated by experimental instructions.

As shown by the interaction of the questionnaires’ rating between stimulation (i.e., synchronous and asynchronous) and type of instructions, the sense of body ownership was significantly stronger when participants judged their *feelings* than their *beliefs* (i.e., items 1 and 2 of the illusion questionnaire). This result is an important sanity check, since while healthy participants report *feeling* like the rubber hand is part of their body, they obviously do not *believe* that it really is.

We have shown that the two classical measures of the RHI (i.e., sense of body ownership vs reallocation of the limb) differ if participants are asked to respond about their *feelings* versus their *beliefs*. These results demonstrate that, in the context of the RHI, how instructions provided by the experimenter, are phrased can significantly bias participants’ responses, that in the present context is reflected in changes in experience body ownership and in turn the embodiment of a rubber hand. These results speak in favour of two different underlying processes for the sense of ownership and the shift in the perceived location of the limb. Previous studies have shown that the sense of ownership and the perceived reallocation of the limb towards the rubber hand are served by different neural mechanisms. For instance, Ehrsson and colleagues [15] proposed that the sense of ownership is mediated by the premotor cortex, whereas a reaching circuit is responsible for the recalibration of the limb position representations. Moreover, a third mechanism responsible for the multisensory integration has been also suggested to underlie this illusion [15]. Our research further supports the idea of the absence of a direct causal link between the sense of body ownership and the proprioceptive drift [7,16]. Importantly, our research also emphasises that the experimenter phrasing to participants when asking about their experience of the illusion should be taken into account in the design and data analyses of all relevant future studies that use the RHI paradigm.
